# Three-Dimensional Analysis of the Effect of Osteosarcoma on Sensory Nerves Innervating the Femur in a Murine Model of Osteosarcoma-Induced Bone Pain

**DOI:** 10.3390/cancers17213533

**Published:** 2025-10-31

**Authors:** John-Paul Fuller-Jackson, Chelsea Hopkins, Jenny Thai, Mie Brandt Lassen, Anne-Marie Heegaard, Jason Ivanusic

**Affiliations:** 1 Department of Anatomy and Physiology, University of Melbourne, Parkville, Melbourne, VIC 3010, Australia; johnpaul.fullerjackson@unimelb.edu.au (J.-P.F.-J.); jenny.thai@unimelb.edu.au (J.T.); 2 Department of Drug Design and Pharmacology, University of Copenhagen, DK-2100 Copenhagen, Denmark; chelsea.hopkins@sund.ku.dk (C.H.); rzm232@sund.ku.dk (M.B.L.); amhe@sund.ku.dk (A.-M.H.)

**Keywords:** bone cancer pain, osteosarcoma, bone pain, pain, sensory nerves, CGRP, NF200

## Abstract

**Simple Summary:**

This study aimed to determine how cancer affects sensory nerve density and distribution, using 3D analysis and mapping tools applied to a murine model of osteosarcoma-induced bone pain. Pain-like behavior, nerve density and distribution were assessed in osteosarcoma bearing and control mice. Comparisons between osteosarcoma bearing and control mice revealed that osteosarcoma differentially affects the density and distribution of different subtypes of peripheral sensory nerves in bone. Understanding how osteosarcomas affect different populations of sensory nerves could lead to more targeted mechanism-based treatments for bone cancer-induced pain.

**Abstract:**

Background: The ways in which peripheral sensory nerves interact with osteosarcomas are important to understand because it could lead to development of new approaches to treat bone cancer pain. This study aimed to determine how cancer affects sensory nerve density and distribution in a murine model of osteosarcoma-induced bone pain. Methods: The femoral marrow cavities of male C3H/HeNHsd mice were injected with either NCTC 2472 primary osteosarcoma (cancer) cells or phosphate buffered saline (control). Pain behavior was assessed using limb use score and static weight bearing assays. At the experimental endpoint, femurs were collected, decalcified, immunolabeled, cleared and imaged using light sheet microscopy (Ultramicroscope Blaze, Miltenyi Biotec). The distribution of sensory nerves was traced through the marrow cavity of the proximal femur and the periosteum overlying the third trochanter (Imaris, Bitplane). Results: Weight bearing on the injected limb was decreased in osteosarcoma-injected but not saline-injected mice. Filament tracing revealed a reduced density of neurofilament 200 kDa-positive (NF200+; myelinated nerve marker) but not calcitonin gene-related peptide-positive (CGRP+; peptidergic nerve marker) sensory nerves in the marrow cavity of osteosarcoma-injected relative to saline-injected mice. There was increased density of CGRP+ but not NF200+ nerves in the periosteum of osteosarcoma-injected relative to saline-injected mice. Conclusions: Osteosarcoma differentially affects the density and distribution of different subtypes of peripheral sensory nerves in bone. Understanding how osteosarcomas affect different populations of sensory nerves could lead to more targeted mechanism-based treatments for bone cancer-induced pain.

## 1. Introduction

Osteosarcomas are aggressive, osteogenic tumors that characteristically involve the long bones of the appendicular skeleton, especially the femur [1,2,3]. Pain is overwhelmingly the most common sign on presentation to the clinical environment and is reported by up to 91% of patients with osteosarcoma [2,3,4]. It typically presents as a dull and diffuse ongoing pain early in disease and becomes more intense and intermittent as disease progresses [5,6]. The mechanisms that generate and maintain osteosarcoma-induced bone pain are not well understood, and this has hampered the development of new approaches to treat it.

A number of animal models of osteosarcoma-induced pain have been used to study pain associated with bone cancer [7,8,9,10,11,12,13,14,15,16,17,18]. The most commonly used model involves direct injection of fibrosarcoma cells (derived from the NCTC 2472 tumor cell line) into the femoral marrow cavity of mice [7]. The tumor cells grow in a highly reproducible fashion as they proliferate and replace hematopoietic cells in the bone marrow. Pain-like behaviors increase in severity with time and are correlated with tumor growth and progressive tumor-induced bone destruction mirroring that which occurs in patients with human osteolytic bone cancer [7,8].

The ways in which peripheral sensory nerves interact with osteosarcomas are important to understand because they could lead to development of mechanism-based approaches to treat bone cancer pain in patients. However, only a few studies have attempted to determine how peripheral sensory nerves interact with osteosarcomas. Some of these reported no difference in the innervation of bone marrow or overlying periosteum in osteosarcoma bearing relative to control animals [7]. Others reported sprouting of nerves in the periosteum and/or skin overlying the tumor [11,13,16,18] or a lack of peripheral sensory nerves in cancer bearing bones [9,10]. Whilst many provided important quantitative and reproducible data on sensory nerve remodeling in bone cancer models, they were all confined to conventional histological analysis of thin sections of bone, and so it is difficult to appreciate and comprehensively analyze dispersed neuronal projections due to the loss or distortion of important structural information when reconstructing 3D images from multiple thin sections. Furthermore, most of the studies reported findings late in disease progression, when there was significant destruction of cortical bone and periosteal involvement [16,19], and none compared different subtypes of peripheral sensory neurons in different parts of the bone early in disease when the cancer was confined mostly to the marrow cavity.

We have recently developed a modified version of the immunolabeling-enabled imaging of solvent-cleared organs (iDISCO/iDISCO+) protocol, optimized for hard tissue, to examine innervation of whole, intact bone [20]. Using this approach, we are now able to label and visualize the nerve terminal endings of different subpopulations of sensory neurons deep in the marrow cavity and the overlying periosteum of the same animals and follow their projections through full 3D volumes of bone. In the present study, we have used this approach to characterize and quantify the full extent of innervation of osteosarcoma bearing bone, by different subpopulations of peripheral sensory neurons, early in disease progression when osteosarcoma is mostly confined to the bone marrow. We have specifically explored roles for two different subtypes of peripheral sensory neurons that are thought to contribute to osteosarcoma-induced bone pain. Peptidergic sensory neurons express calcitonin gene-related peptide (CGRP) and are predominantly small diameter, unmyelinated (C) neurons that are likely to mediate slow, dull and diffuse pain of skeletal origin [21,22,23,24,25]. Myelinated sensory neurons express neurofilament 200 kDa (NF200), which is confined to small diameter myelinated (Aδ) neurons in bone, and are likely mediate fast pain of skeletal origin [22,24,25,26,27].

## 2. Materials and Methods

### 2.1. Animals

Twenty-one six-week-old, male C3H/HeNHsd (Inotiv, Lafayette, IN, USA) and two male C57Bl6 mice were used in this study. Twelve C3H/HeNHsd mice were injected with NCTC 2472 cells and nine underwent sham surgery with phosphate buffered saline (PBS) injected into the femur. Three were excluded from the study because they did not develop a tumor in the bone (established by X-ray imaging). The two C57Bl6 mice were used to characterize the NF200 antibody. Animals were group-housed in a 12 h light/dark cycle and were provided with food and water ad libitum. Live animal experiments were conducted according to the Danish Animal Experiments Inspectorate (Copenhagen, Denmark, 2020_15_0201_00439) in a specific pathogen-free facility at the University of Copenhagen (Materials and Methods, osteosarcoma vs. sham control animals) or were approved by the University of Melbourne Animal Care and Ethics Committee and conducted at the University of Melbourne (Supplementary Methods, animals used to characterize antibodies).

### 2.2. Tumor Cells

NCTC 2472 sarcoma cells (CCL-11, American Type Culture Collection, Manassas, VA, USA) were cultured for two weeks prior to surgery at 37 °C in NCTC-135 medium (Gibco, Carlsbad, CA, USA) with 10% horse serum (Gibco, Carlsbad, CA, USA, Lot: 2593036). They were passaged at 80% confluency, two days prior to surgery and on the day of surgery, by applying 0.05% trypsin-EDTA (Gibco, Carlsbad, CA, USA) and incubating for four minutes at 37 °C. For surgery, cells were resuspended in 0.1 M PBS at 10^7^ cells/mL.

### 2.3. Surgery

Surgery was conducted as previously described [28]. In brief, C3H/HeNHsd mice were anesthetized with ketamine/xylazine (43 mg/kg Ketaminol, MSD Animal Health, Boxmeer, The Netherlands; 6 mg/kg Xylazine, Rompun Vet, Bayer, Germany) and supplemented with 1–1.2% isoflurane (Attane Vet, ScanVet, Fredensborg, Denmark). Carprofen (5 mg/kg Carprosan Vet, Dechra, Leverkusen, North Rhine-Westphalia, The Netherlands) was injected subcutaneously prior to surgery (Day 0) and on Day 1 post-surgery. A small skin incision was made at the knee to identify the patellar tendon, which was displaced laterally to expose the distal femur. A 0.3 mL insulin syringe (BD, Franklin Lakes, NJ, USA) was used to inject 10 µL of NCTC 2472 cells (osteosarcoma group) or 0.1 M PBS (control group). The syringe was maintained in position for 1 min before being removed, and the hole was filled with bone wax (Harvard Apparatus, Holliston, MA, USA). The skin incision was closed with surgical clips (Michel Suture Clips, Agnthos, Lidingö, Sweden).

### 2.4. Experimental Endpoint

**Figure 1:**
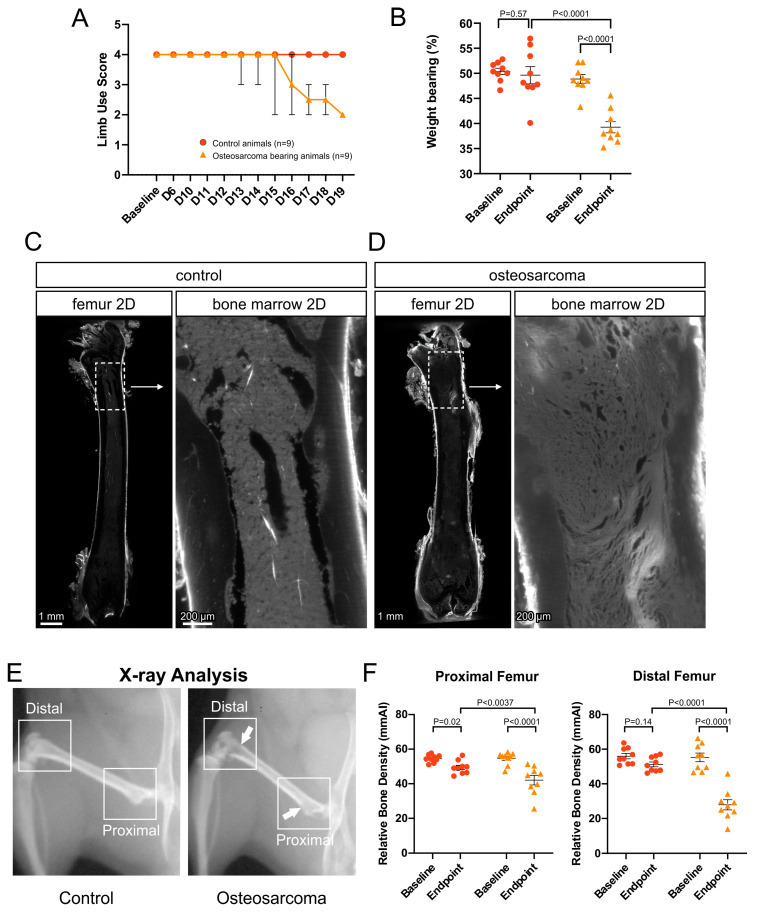
Pain behavior and tumor growth. (**A**) Limb use score following intrafemoral injection of phosphate buffered saline (PBS; control animals) or NCTC 2472 cells (osteosarcoma bearing animals). Data are presented as the median ± range. (**B**) Percentage of weight bearing on the injected hindlimb, at baseline and experimental endpoint, in control vs. osteosarcoma bearing animals. Data presented as the mean ± standard error of the mean, two-way ANOVA with Fisher’s LSD. (**C**) Low power (4×) image of a 40 µm z-projection of the whole femur (left) and marrow cavity (inset) from a control animal. The marrow cavity had a normal appearance. (**D**) Low power (4×) image of a 40 µm z-projection of the whole femur (left) and marrow cavity (inset) from an osteosarcoma bearing animal. By the endpoint, osteosarcoma cells had proliferated and replaced hematopoietic cells in the entire marrow cavity and had begun to impact on cortical bone but did not break through to the periosteum. (**E**) Radiographs of hindlimbs from control and osteosarcoma bearing animals. Arrowhead indicates an area of reduced bone density. (**F**) Relative bone density of the femurs from control and osteosarcoma bearing animals at baseline and endpoint. Data presented as the mean ± standard error of the mean, two-way ANOVA with Fisher’s LSD. Red circles represent control animals, orange triangles represent osteosarcoma bearing animals.

The experimental endpoint for osteosarcoma-injected animals was defined as a limb use score of 2, when significant pain-like behaviors (limb use and weight bearing) have developed but tissue damage is not extensive. This was chosen because we were interested in understanding what happens to the nerves in subchondral bone early in disease progression before the tumor breaks through bone to impact on the periosteum. Previous analysis of tumor growth in this model using GFP fluorescence and H&E sections revealed that by Days 14–17 post-injection of NCTC cells, the tumor completely occupies the femoral marrow cavity [12,29]. This coincides broadly with when most of the animals reported in the present study reached a limb use score of 2 (Figure 1A). The experimental endpoint for sham animals was at Day 19 (the day of the last osteosarcoma-injected animal to reach a limb use score of 2).

### 2.5. Behavioral Tests

Pain behavior was assessed using a limb use score and static weight bearing assay. Testing was always performed at the same times each day. Animals were habituated to the testing equipment each day on Days −6 to −2. Behavioral tests were conducted on Day −1 (baseline), Day 6 (after recovery from surgery), every day from Day 10–14 and thereafter only every second day unless they demonstrated a limb use score of 3, after which they were assessed daily until the experimental endpoint. The last weight bearing data point available was carried forward if animals reached the experimental endpoint on a day that static weight bearing was not assayed.

To determine the limb use score, mice were placed into an empty transparent box where they were monitored for 3 min. The following scale was used to assess their gait:

4—Normal gait.

3—Insignificant limping.

2—Significant limping and shift in bodyweight towards the healthy limb.

1—Significant limping and partial lack of use of the ipsilateral leg.

0—Total lack of use of the ipsilateral leg.

Static weight bearing was assayed using an Incapacitance Tester (version 5.2, Linton, UK), which independently measures weight bearing on each hindlimb. At each testing time point, the average of 3 weight bearing readings of 3 s in duration was calculated, and the weight borne on the injected hindlimb limb was expressed as a percentage of the total weight bearing on both hindlimbs.

### 2.6. X-Ray Imaging

Mice were anesthetized with 2–2.5% isoflurane (Attane Vet, ScanVet, Fredensborg, Denmark), and a Lumina XR Apparatus In Vivo Imaging System (IVIS, Revitty, Belgium; exposure settings: 28 s, 35 kVp) was used to obtain X-rays of their hindlimbs at baseline and the experimental endpoint. By including an aluminum step wedge in the radiograph, the varying shades of gray in the bone can be compared to the shades of the aluminum wedge, allowing for a conversion of the bone’s density into an equivalent thickness of aluminum (mmAl). In this study, we report relative bone density measured using FIJI (Version 1.54p, NIH, Bethesda, MD, USA). The mean grayscale value of a standard region of interest was measured within the proximal or distal femur, and the mean of two additional grayscale values acquired from adjacent soft tissue regions was subtracted from the grayscale values of each region of interest. The resulting value was adjusted to an aluminum step wedge imaged with the bone using the following equation: (Grayscale value of wedge × (Grayscale value of Femur − Mean grayscale value soft tissue))/100.

### 2.7. Perfusion Fixation and Tissue Collection

At the experimental endpoint, mice were anesthetized with ketamine/xylazine (85.5 mg/kg Ketaminol, MSD Animal Health, Boxmeer, the Netherlands; 12 mg/kg Xylazine, Rompun vet, Bayer, Leverkusen, North Rhine-Westphalia, Germany) and perfuse-fixed via intracardiac perfusion with 30–40 mL of PBS, followed by 30–40 mL of chilled 4% paraformaldehyde (PFA; Sigma-Aldrich, Burlington, MA, USA). The femur was removed and immerse-fixed in 4% PFA at 4 °C overnight, washed 3 times with 0.1 M PBS, then wrapped in gauze soaked with 0.1 M PBS and 0.1% sodium azide (Sigma Aldrich, Burlington, MA, USA) and shipped to the University of Melbourne in Australia.

### 2.8. Tissue Clearing

Samples were processed using the tissue clearing protocol we have previously described [20]. Briefly, femurs were washed three times in PBS and decalcified in Morse’s solution for one week at room temperature. After decalcification, they were washed six times in 1× Dulbecco’s phosphate buffered saline (DPBS; Gibco, Waltham, MA, USA), dehydrated in 50%, 80% and 100% methanol and bleached in 6% hydrogen peroxide in methanol overnight. They were rehydrated in 100%, 100%, 80% and 50% methanol in DPBS, then in DPBS alone and were blocked in DPBS with gelatin, 0.5% Triton X-100 and 0.1% thimerosal (DPBSG-T). Immunolabeling was achieved with incubation in rabbit anti-CGRP (Sigma-Aldrich, St. Louis, MO, USA, #C8198, 1:1000 dilution) or rabbit anti-NF200 (Sigma-Aldrich, St. Louis, MO, USA, #N4142, 1:1000 dilution) primary antibodies for 11 days, followed by washing in DPBS with 0.5% Triton X-100 (DPBS-T), and further incubation in donkey anti-rabbit Alexa Fluor-647 antibody (Invitrogen, Waltham, MA, USA, #A31573, 1:1000 dilution) for 7 days. The samples were washed again in DPBS-T, dehydrated in 20%, 40%, 60%, 80%, 100% and 100% methanol and incubated overnight in 66% dichloromethane (DCM; Sigma-Aldrich, St. Louis, MO, USA) and 33% methanol. The following day, samples were incubated three times in DCM and cleared in dibenzyl ether (DBE; Sigma-Aldrich, St. Louis, MO, USA) before being transferred to ethyl cinnamate (ECi; Sigma-Aldrich, St. Louis, MO, USA) for imaging.

### 2.9. Antibody Specificity and Characterization

The specificity of the primary antibodies we used for CGRP and NF200 immunolabeling has been reported in our previous studies [20,22]. CGRP is the most common marker used to identify peptidergic sensory neurons in bone [21,30,31,32,33,34]. NF200 is a neurofilament protein that labels myelinated sensory neurons in mice, and it is confined to small diameter, presumed Aδ nociceptive neurons in bone [22,24]. NF200 immunolabeling in murine bone is distinct from the corkscrew appearance of tyrosine hydroxylase labeled sympathetic nerve endings around blood vessels in bone [20,22], and transcriptomic analysis of nefh (the gene for the heavy chain neurofilament also known as NF200) reveals no expression in sympathetic compared to sensory neurons in rats [35]. In the present study, we further confirmed NF200 protein expression in murine sensory, but not sympathetic, neurons, using C57Bl6 mice available at the University of Melbourne (Supplementary Methods, Figure S1).

### 2.10. Imaging

**Figure 2:**
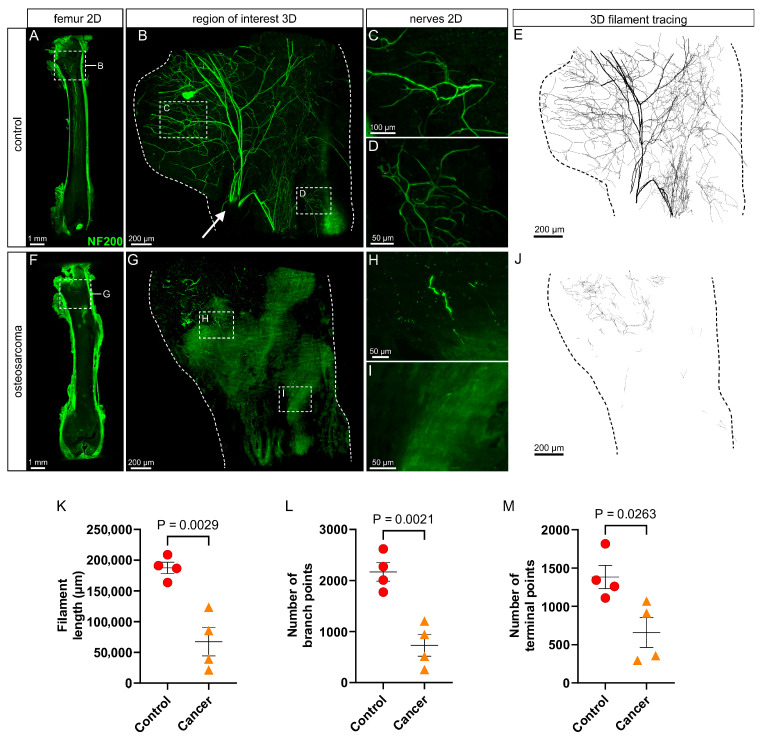
Neurofilament 200 kDa (NF200)-immunolabeled nerve profiles in the marrow cavity of control and osteosarcoma bearing animals. (**A**) Low power (4×) image of a 200 µm z-projection of the femur showing NF200+ nerves in the marrow cavity of a control animal, and the region of interest that analysis was applied to (inset, (**B**)). (**B**) High power (12×) image of a 1500 µm z-projection, through the full thickness of the marrow cavity, showing NF200+ nerves in the region of interest. A 3D projection of these nerve profiles is available as a video (Supplementary Data, nf200-control.mp4). Arrow indicates primary nerve entry point in region of interest. (**C**,**D**) Insets show 50 µm z-projections of NF200+ nerve fibers indicated in (**B**). (**E**) Full thickness z-projection of NF200+ nerve filaments traced from (**B**). (**F**) Low power (4×) image of a 200 µm z-projection of the femur showing NF200+ nerves in the marrow cavity of an osteosarcoma bearing animal, and the region of interest that analysis was applied to (inset, (**G**)). (**G**) High power (12×) image of a 1500 µm z-projection through the full thickness of the marrow cavity, showing NF200+ nerves in the region of interest. A 3D projection of these nerve profiles is available as a video (Supplementary Data, nf200-cancer.mp4). (**H**,**I**) Insets show 50 µm z-projections of NF200+ nerve fibers indicated in (**G**). (**J**) Full thickness z-projection of NF200+ nerve filaments traced from (**G**). There was a reduction in (**K**) filament length, (**L**) number of branch points, and (**M**) number of terminal points in osteosarcoma bearing (*n* = 4) relative to control (*n* = 4) animals. Data presented as the mean ± standard error of the mean, unpaired *t*-test.

**Figure 3:**
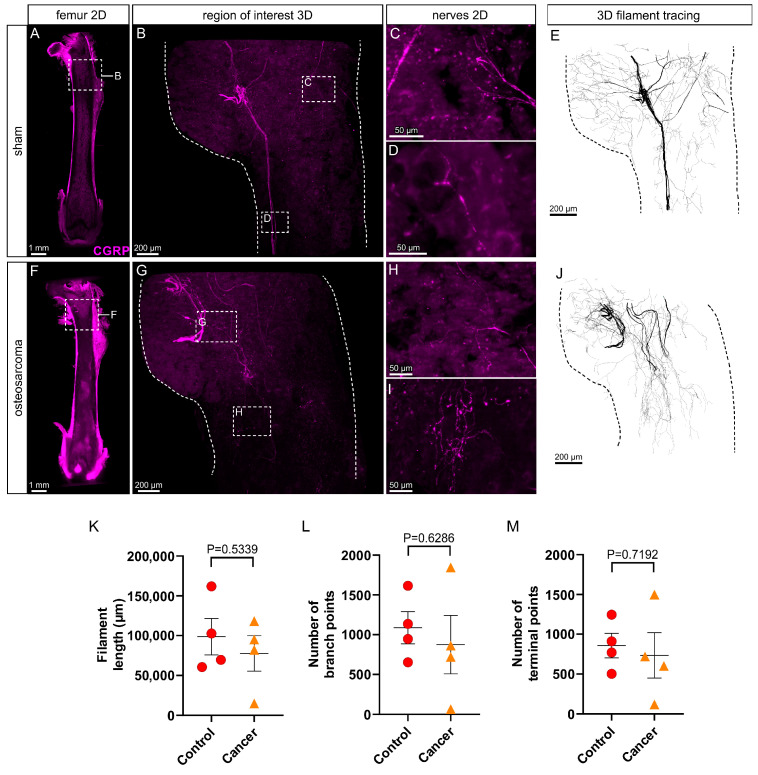
Calcitonin gene-related peptide (CGRP) immunolabeled nerve profiles in the marrow cavity of control and osteosarcoma bearing animals. (**A**) Low power (4×) image of a 200 µm z-projection of the femur showing CGRP+ nerves in the marrow cavity of a control animal and the region of interest that analysis was applied to (inset, (**B**)). (**B**) High power (12×) image of a 1500 µm z-projection, through the full thickness of the marrow cavity, showing CGRP+ nerve profiles in the region of interest. A 3D projection of these nerve profiles is available as a video (Supplementary Data, cgrp-control.mp4). (**C**,**D**) Insets show 50 µm z-projections of CGRP+ nerve fibers indicated in (**B**). (**E**) Full thickness z-projection of CGRP+ nerve filaments traced from (**B**). (**F**) Low power (4×) image of a 200 µm z-projection of the femur showing CGRP+ nerves in the marrow cavity of an osteosarcoma bearing animal, and the region of interest that analysis was applied to (inset, (**G**)). (**G**) High power (12×) image of a 1500 µm z-projection through the full thickness of the marrow cavity, showing CGRP+ nerves in the region of interest. A 3D projection of these nerve profiles is available as a video (Supplementary Data, cgrp-cancer.mp4). (**H**,**I**) Insets show 50 µm z-projections of CGRP+ nerve fibers indicated in (**G**). (**J**) Full thickness z-projection of CGRP+ nerve filaments traced from (**G**). There was no change in (**K**) filament length, (**L**) number of branch points and (**M**) number of terminal points in osteosarcoma bearing (*n* = 4) relative to control (*n* = 4) animals. Data presented as the mean ± standard error of the mean, unpaired *t*-test.

Cleared samples were imaged using a light sheet microscope (Ultramicroscope Blaze, Miltenyi Biotec, Bergisch Gladbach, Germany) with a Zyla sCMOS camera (Andor, Belfast, UK) and a 4×/0.35 or 12×/0.53 objective lens. Optical z-stacks were generated using a step size of 2 µm with 50 ms exposure per step. Mosaic acquisitions were performed with 10% overlap. Images were captured using ImSpector Pro software (version 7.6.3, Miltenyi Biotec, Bergisch Gladbach, Germany). Image stacks were converted to Imaris format, stitched and visualized using Imaris software (version 10.2.0, Bitplane, Belfast, UK). Tracing of nerve profiles and axons was performed using the Imaris “Filament Tracer” module. For tracing of nerve profiles in the marrow cavity, a consistent region of interest was chosen to include the proximal femur, surrounding the entry of the main nerve through the nutrient foramen (3629 × 3651 µm^2^, full thickness of bone, 12× magnification, Figure 2A,F and Figure 3A,F), as this is the most densely innervated part of the murine femur [20]. For tracing nerve profiles and axons in the periosteum, a consistent region of interest was chosen on the posterior aspect of the third trochanter (851 × 991 µm^2^, full thickness of periosteum, 12× magnification, Figure S2). Tracing was performed manually through the entire image stack in each region of interest, with careful attention to tracing clearly identifiable nerves and axons, which were easily distinguished from surrounding structures, like blood vessels, that were not labeled. Filament length, number of filament branches and number of filament terminals were exported for quantitative analysis. All figures were prepared as 3D images or maximum intensity projections on Imaris. Movies were generated in Imaris with a frame rate of 24 frames per second. Figures were constructed in Corel Draw software (version 24.3.0.571, CorelDRAW Graphics Suite, Corel Corporation, Ottawa, ON, Canada). Only small linear adjustments to brightness and contrast were made to the figures.

### 2.11. Statistics

Weight bearing data and relative bone density were assessed using two-way ANOVA with repeated measures, followed by Fisher’s LSD test only if the ANOVA was significant. Comparisons of filament length, filament branching and number of terminals were made using unpaired *t*-tests. All statistical comparisons and graphs were made using GraphPad Prism (version 10.4.1, GraphPad Software, San Diego, CA, USA). *p* < 0.05 was considered statistically significant. Whilst the findings for quantitative analysis of filament data should be interpreted in the context of a small sample size (*n* = 4 per group), the data passed tests for normality (Shapiro–Wilk test) and equal variance (F test).

## 3. Results

### 3.1. Osteosarcoma-Induced Pain Behavior and Tumor Growth

The experimental endpoint (limb use score of 2) was reached as early as Day 13 in some osteosarcoma-injected animals (Figure 1A). There was a clear reduction in limb use in the majority of osteosarcoma animals from Days 16–19 (Figure 1A). In contrast, there was no change in limb use at any timepoint in control animals (Figure 1A). All osteosarcoma-injected animals had decreased weight bearing at the experimental endpoint (Figure 1B). Two-way ANOVA revealed a significant interaction effect for weight bearing (F [1, 16] = 22.37, *p* = 0.0002). Weight bearing at the experimental endpoint was significantly reduced in osteosarcoma-injected relative to control animals (Fishers LSD, *p* < 0.0001, Figure 1B). It was also significantly reduced relative to baseline in osteosarcoma-injected (Fishers LSD, *p* < 0.0001, Figure 1B), but not in control, animals (Fishers LSD, *p* = 0.5748, Figure 1B). At the experimental endpoint, control animals had a well-organized, normal femoral marrow cavity appearance (Figure 1C). In contrast, the entire femoral marrow cavity of osteosarcoma-injected animals was less organized and devoid of normal morphology, consistent with tumor growth through the whole bone (Figure 1D). X-ray analysis revealed a reduction in bone density in osteosarcoma-injected compared to control animals (Figure 1E,F). Two-way ANOVA revealed a significant interaction effect for relative bone density both in the proximal (F [1, 16] = 7.792, *p* = 0.0131) and distal (F [1, 16] = 20.94, *p* = 0.0003) femur. At the experimental endpoint, there was a significant reduction in relative bone density in the proximal (Fishers LSD, *p* = 0.0044, Figure 1F) and distal (Fishers LSD, *p* < 0.0001, Figure 1F) femur of osteosarcoma-injected compared to control animals. Relative bone density at the experimental endpoint was also significantly reduced compared to baseline in both the proximal (Fishers LSD, *p* < 0.0001, Figure 1F) and distal (Fishers LSD, *p* < 0.0001, Figure 1F) femur of osteosarcoma-injected animals.

### 3.2. There Is a Marked Loss of Myelinated (NF200+) Nerve Profiles in the Marrow Cavity of Osteosarcoma Bearing Relative to Control Animals

In control animals, there was extensive innervation of the femur by myelinated (NF200+; Figure 2A–E) sensory nerves. In general, NF200+ nerve bundles entered the femoral marrow cavity via nutrient foramina located at the proximal metaphysis (e.g., arrow in Figure 2B). The nerves branched extensively into the trochanter and through the neck of the femur above and into the marrow cavity of the diaphysis below (Figure 2B,E). The branches that ran down through the diaphysis often ran in parallel and terminated over long distances, some extending all the way down to the distal metaphysis (Figure 2A). Quantification by filament tracing revealed the average length of filament, number of branch points and number of endings of myelinated (NF200+) nerve profiles in the proximal femur of control animals were 187,500 ± 18,602 µm, 2168 ± 364 and 1383 ± 305, respectively (Figure 2K–M). A 3D video that captures the full extent and typical appearance of NF200+ nerves in the proximal femur of a control animal is presented as Supplementary Materials (nf200-control.mp4).

There was a clear reduction in the innervation of the proximal femur of osteosarcoma-injected animals by myelinated (NF200+) nerves (Figure 2F–J). Axons and nerve terminal endings were distributed sparsely throughout the femoral marrow cavity (Figure 2G,J), mostly appearing as small fragments of discontinuous axon (Figure 2H,I). Filament length (67,341 ± 46,204 µm, *p* = 0.0029), number of branch points (729 ± 425, *p* = 0.0021) and number of endings (658 ± 389, *p* = 0.0263) were all significantly reduced in osteosarcoma-injected relative to control animals (unpaired *t*-tests, Figure 2K–M). A representative 3D video of NF200+ immunolabeling in the proximal femur of an osteosarcoma-injected animal is presented as Supplementary Data (nf200-cancer.mp4).

### 3.3. There Is No Change in Peptidergic (CGRP+) Innervation in the Marrow Cavity of Osteosarcoma Bearing Relative to Control Animals

In control animals, there was extensive innervation of the femur by peptidergic (CGRP+; Figure 3A–E) sensory nerves. The pattern of innervation by CGRP+ nerves was similar to that of NF200+ nerves. CGRP+ nerve bundles entered the femoral marrow cavity via nutrient foramina located at the proximal metaphysis and branched extensively into the trochanter and through the neck of the femur above and into the marrow cavity of the diaphysis below (Figure 3B,E), often extending all the way down to the distal metaphysis (Figure 3A). Quantification by filament tracing revealed the average length of filament, number of branch points and number of endings of peptidergic (CGRP+) nerve profiles in the proximal femur of control animals were 98,565 ± 45,931 µm, 1087 ± 404 and 857 ± 309, respectively (Figure 3K–M). A 3D video that captures the full extent and typical appearance of CGRP+ nerves in the proximal femur of a control animal is presented as Supplementary Materials (cgrp-control.mp4).

In contrast to myelinated (NF200+) sensory nerves, there were no obvious changes to peptidergic (CGRP+) nerves in the proximal femur of osteosarcoma-injected animals (Figure 3F–J). Whilst these animals all had decreased weight bearing at the endpoint (Figure 1B), the morphology and distribution of CGRP+ nerves appeared similar to that in control animals. Filament length (77,501 ± 44,362 µm, *p* = 0.5339), number of branch points (873 ± 734, *p* = 0.6286) and number of endings (734 ± 572, *p* = 0.7192) were all unchanged in osteosarcoma-injected relative to control animals (unpaired *t*-tests, *p* > 0.05, Figure 3K–M). A representative 3D video of CGRP+ immunolabeling in the proximal femur of an osteosarcoma-injected animal is presented as Supplementary Data (cgrp-cancer.mp4).

### 3.4. There Is an Increase in Peptidergic (CGRP+) but Not Myelinated (NF200+) Nerve Profiles in the Periosteum Overlying Bone in Osteosarcoma Bearing Relative to Control Animals

**Figure 4:**
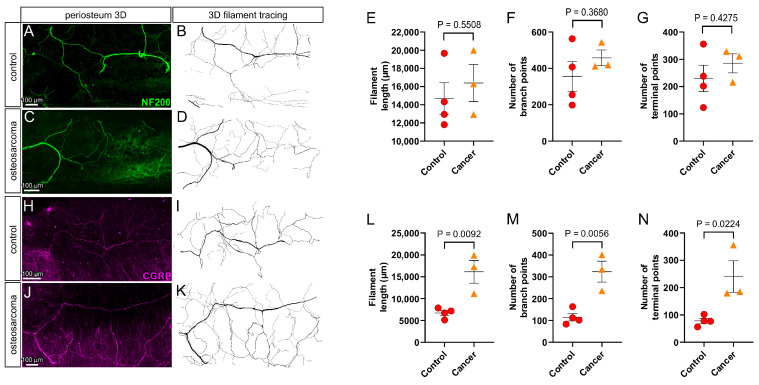
Sprouting of calcitonin gene-related peptide (CGRP) but not neurofilament 200 kDa (NF200) nerves in the periosteum of osteosarcoma bearing animals. (**A**,**C**,**H**,**J**) Full thickness z-projections of high power (12×) images of nerves in the periosteum overlying the posterior aspect of the third trochanter. (**B**,**D**,**I**,**K**) Full thickness z-projections of nerve filaments traced from (**A**,**C**,**H**,**J**). There was no difference in (**E**) filament length, (**F**) number of branch points and (**G**) number of terminal points of NF200+ nerve profiles in osteosarcoma bearing (*n* = 4) relative to control (*n* = 4) animals. There was an increase in (**L**) filament length, (**M**) number of branch points and (**N**) number of terminal points of CGRP+ nerve profiles in osteosarcoma bearing (*n* = 4) relative to control (*n* = 4) animals. Data presented as the mean ± standard error of the mean, unpaired *t*-test.

In control animals, there was extensive myelinated (NF200+; Figure 4A,B) and peptidergic (CGRP+; Figure 4H,I) innervation of the periosteum overlying the proximal femur. Quantification by filament tracing revealed the average length of filament, number of branch points and number of endings of myelinated (NF200+) and peptidergic (CGRP+) nerve profiles in the proximal femur of control animals were NF200: 14,689 ± 1737 µm (length), 356 ± 82 (branch points) and 230 ± 48 (endings) (Figure 4E–G), and CGRP: 6719 ± 584 µm (length), 115 ± 17 (branch points) and 78 ± 9.4 (endings) (Figure 4L–N), respectively. There was a significant increase in filament length (16,126 ± 2604 µm, *p* = 0.0092), number of branch points (324 ± 83, *p* = 0.0056) and number of endings (240 ± 58, *p* = 0.0224) of peptidergic (CGRP+) nerve profiles in the periosteum of osteosarcoma-injected relative to control animals (unpaired *t*-tests, Figure 4J–N). In contrast, there was no difference in filament length (16,396 ± 2039 µm, *p* = 0.5508), number of branch points (458 ± 43, *p* = 0.3680) and number of endings (285 ± 35, *p* = 0.4275) of myelinated (NF200+) nerve profiles in the periosteum of osteosarcoma-injected relative to control animals (unpaired *t*-tests, Figure 4C–G).

## 4. Discussion

The main finding of the present study is that osteosarcoma differentially affects different subpopulations of peripheral sensory neurons that innervate cancer bearing bones. There was a marked reduction in NF200+ but not CGRP+ nerve profiles in the femoral marrow cavity of osteosarcoma bearing relative to control animals, suggesting that neuropathy of myelinated but not peptidergic sensory neurons in the marrow cavity contributes to osteosarcoma-induced pain. There was also an increase in CGRP+ but not NF200+ nerve profiles in the periosteum of osteosarcoma bearing relative to control animals, suggesting sprouting of periosteal peptidergic but not myelinated sensory neurons also contributes to osteosarcoma-induced pain.

There are only a few studies investigating how osteosarcoma affects nerves in cancer bearing bone. In the earliest study, occasional CGRP+ nerve fibers were reported in NCTC 2472 tumor masses in the marrow cavity of bone, and there were no obvious changes in the periosteum [7]. However, the authors did not show any data to support their findings for the marrow cavity, and they acknowledged that destruction of both mineralized bone and periosteum at the late stage of disease they studied made it difficult to quantify changes. A later study by the same group reported that the marrow cavity of osteosarcoma bearing bone was devoid of both NF200+ and CGRP+ nerve fibers but showed only one histological section, and they did not quantify data, making it difficult to appreciate the full extent of the change they asserted [9]. In the present study, we were able to resolve the full extent of myelinated and peptidergic innervation of bones and use filament tracing to carefully quantify differences between cancer bearing and control animals. Our findings clearly show that osteosarcoma differentially affects specific subpopulations of sensory nerves that innervate bone and that the effect varies between the marrow cavity and periosteum.

Whilst bone cancer pain is driven by combinations of nociceptive, inflammatory and/or neuropathic processes [36], the precise way by which these processes interact with nociceptive neurons to elicit pain remains unknown. In the present study, we have clearly shown marked neuropathy of myelinated (NF200+) sensory nerves in the marrow cavity of osteosarcoma bearing bone relatively early in disease progression. The neuropathy is likely due to destruction and/or injury of the axons and nerve terminal endings by the tumor, which is predominantly osteolytic. This is consistent with reports of increased expression of ATF3 (a marker for neuronal injury) in peripheral sensory neurons of osteosarcoma bearing compared to control animals [9] and reports that osteosarcomas sampled from human bone lack a distinct nerve supply [37,38]. It will be interesting in the future to determine if ATF3 expression or other markers of neuronal injury are altered at this early time point in disease progression in the model we used. Nonetheless, this important finding suggests drugs that specifically target neuropathic pain may be useful to treat osteosarcoma-induced pain. Indeed, treatment with gabapentin attenuates both ongoing and movement-evoked pain behaviors in the same animal model of osteosarcoma-induced pain we have used in this study [9,39] and in humans with osteosarcoma [40] or other bone cancers [41]. Neuropathic pain also occurs after amputation and limb sparing surgery in patients with osteosarcoma [42], and in response to chemotherapy [43], and so this approach may also be relevant to minimizing post-surgical and chemotherapy-induced cancer pain in these patients.

We have also shown that there is sprouting of CGRP+ but not NF200+ nerves in the periosteum overlying osteosarcoma bearing bone. Neuronal sprouting has been reported in the periosteum in animal models of osteosarcoma-induced pain [16,17]. Specifically, both NF200+ and CGRP+ nerves have been reported to sprout in periosteum overlying osteosarcoma bearing bone late in disease progression, once the sarcoma cells begin to break through bone and impact on the periosteum [16,17]. It is possible that we did not observe osteosarcoma-induced changes in NF200+ labeling in the periosteum in the present study because we sampled tissue earlier in disease progression. Nonetheless, our findings indicate that the two different subpopulations we studied were differentially affected, and so they may at least each respond in different ways at different time points of disease progression. The sprouting in the periosteum may be in response to tumor derived factors like NGF released by cells in the tumor–bone microenvironment [16,19]. This may also differentially affect some subpopulations over others. Interestingly, blocking NGF signaling can reduce sprouting in this same model and has been proposed as an alternative approach to pain management for osteosarcoma-induced pain [16,44]. It will be interesting to test whether sequestering NGF can also reduce sprouting early in disease progression, as reported in this study.

The overwhelming majority of sensory neurons that innervate bone are Aδ and C-fiber neurons that express NF200 and/or CGRP [21,23,45,46]. Electrophysiological recordings of bone afferent neurons reveal Aδ bone afferent neurons are relatively fast conducting and have high thresholds for mechanical activation [45] and these are likely small diameter myelinated neurons labeled with antibodies directed against NF200. In contrast, C bone afferent neurons are slow conducting, have high thresholds for mechanical activation and can be activated by known algesic substances, including neurotrophic factors [47,48,49,50]. They are typically small diameter unmyelinated neurons that can be either peptidergic or non-peptidergic. The peptidergic population is labeled with antibodies directed against CGRP [24]. Thus, the NF200+ neurons reported in the present study are likely to be Aδ bone afferent neurons that signal noxious mechanical sensitivity, and the CGRP+ neurons reported in the present study are likely to be peptidergic C bone afferent neurons that function as polymodal nociceptors. Our findings suggest that osteosarcoma-induced mechanical hypersensitivity early in disease progression may in part be driven by changes in small diameter, myelinated Aδ bone afferent neurons that innervate the marrow cavity and/or by changes in small diameter, unmyelinated, peptidergic C-fiber bone afferent neurons that innervate the periosteum. It is likely that this changes later in disease progression because both NF200+ and CGRP+ nerves have been reported to sprout in periosteum overlying osteosarcoma bearing bone once the sarcoma cells begin to break through bone and impact on the periosteum [16,17].

## 5. Conclusions

Pain profiles differ for patients with different types of bone cancer and in different animal models of bone cancer pain. It will be important in the future to determine how the different subpopulations of nerves, in each of the different bony compartments, change with disease progression and if this differs for the different types of cancer (osteolytic vs. osteoblastic) and in males vs. females. Understanding this is important because it could lead to development of mechanism-based treatments to manage bone cancer pain and to targeting the appropriate approach to pain management, at the right time in disease progression, for individual patients.

## Data Availability

Data available from corresponding author upon reasonable request.

## Supplementary Materials

The following supporting information can be downloaded. Supplementary Methods: Characterization of NF200 antibody in sensory and sympathetic ganglia https://www.mdpi.com/article/10.3390/cancers17213533/s1; Video S1: https://doi.org/10.5281/zenodo.17489773, nf200-control, nf200-cancer, cgrp-control, cgrp-cancer.

## Abbreviations

The following abbreviations are used in this manuscript:

NF200	Neurofilament 200 kDa
CGRP	Calcitonin gene-related peptide
PBS	Phosphate buffered saline
mmAl	Millimeter aluminum equivalents
DCM	Dichloromethane
DBE	Dibenzyl ether
ECi	Ethyl cinnamate
