# Supplementary material for: Three-Dimensional Analysis of the Effect of Osteosarcoma on Sensory Nerves Innervating the Femur in a Murine Model of Osteosarcoma-Induced Bone Pain
Source: Cancers (Basel). 2025 Oct 31;17(21):3533. doi: 10.3390/cancers17213533 (PMC12606752; doi:10.3390/cancers17213533)
Supplement: Supplementary file 1 [file cancers-17-03533-s001.zip › cancers-3884022-supplementary.pdf]

Supplementary Materials

Supplementary Methods: Characterization of NF200 antibody in sensory and sympathetic ganglia

Mice were anaesthetized and perfused via the left ventricle with PBS followed by 4% PFA. The entire lumbar vertebral column was removed and decalcified in 10% EDTA for one week. The sample was washed in PBS and placed overnight in 30% sucrose-PBS. It was frozen in liquid nitrogen-cooled isopentane and sectioned at 40 μm using a cryostat. Sections were washed in PBS, blocked in 10% normal horse serum for 1 hr, and incubated overnight with rabbit anti-NF200 (Sigma-Aldrich, #N4142, 1:1000) or rabbit anti-TH (Millipore, #AB152, 1:1000) antibody at room temperature. Sections were washed in PBS, incubated in donkey anti-rabbit Alexa Fluor-647 antibody (Invitrogen, #A31573, 1:200 dilution) for 2 hrs, and washed again in PBS. The slides were cover-slipped using DAKO fluorescence mounting media. Lumbar sympathetic chain ganglia (SCG) and dorsal root ganglia (DRG) were imaged via confocal microscopy (LSM900, Carl Zeiss).

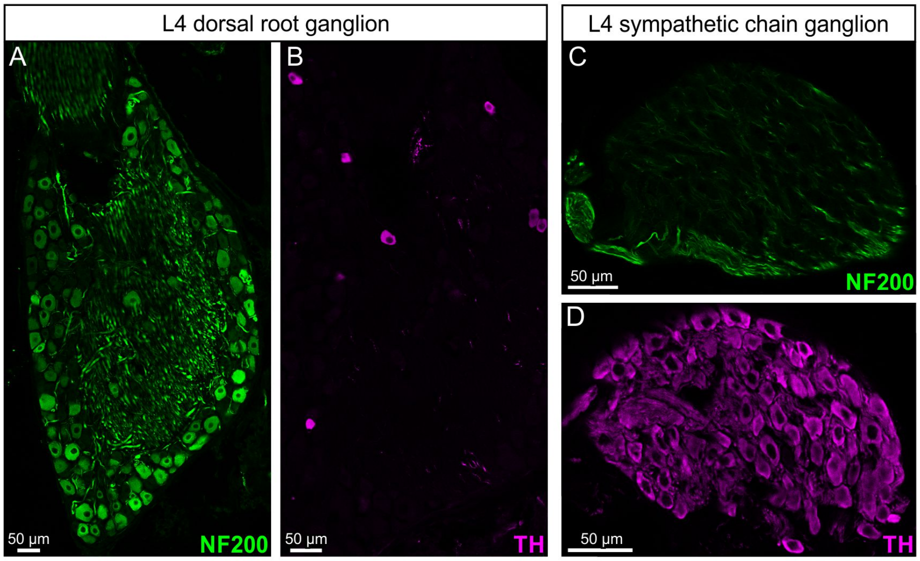

Figure S1. Neurofilament 200 kDa (NF200) is expressed in sensory neurons of the L4 dorsal root ganglia but not the sympathetic neurons in the sympathetic chain ganglion of mice. (A) NF200- and (B) TH-immunolabelling in a cryosection of the L4 dorsal root ganglion. (C) NF200- and (D) TH- immunolabelling in a cryosection of the L4 sympathetic chain ganglion.

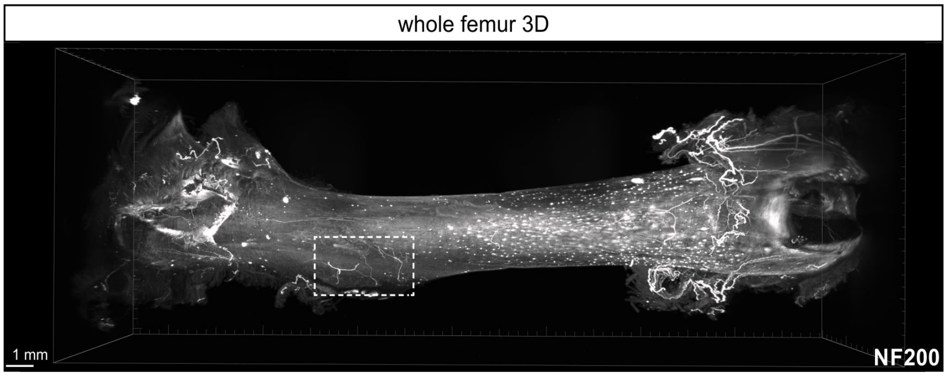

Figure S2. Region of interest for analysis of periosteal filament tracing on the posterior aspect of the third trochanter. 3D visualization of the whole femur immunolabeled for neurofilament 200 kDa (NF200), cleared and imaged via light sheet microscopy at (4x) magnification, with the region of interest on the periosteum indicated by the dotted box.
